# Evaluation of Halitosis, Salivary Flow Rate, and Whole-Saliva pH Across Menstrual Cycle Phases in Healthy Female Dental Students: A Clinical Observational Study

**DOI:** 10.3290/j.ohpd.c_2855

**Published:** 2026-11-06

**Authors:** Sena Ayyıldız, Eda Gürsu Şahin, Başak Karasu

**Affiliations:** a Sena Ayyıldız Assistant Professor, Department of Pediatric Dentistry, Faculty of Dentistry, Çankırı Karatekin University, Çankırı, Turkey. Conceived and designed the study, developed the study hypothesis, coordinated the study procedures, participated in data collection, interpreted the findings, and drafted the manuscript. Reviewed and approved the final version of the manuscript.; b Eda Gürsu Şahin Assistant Professor, Department of Endodontics, Faculty of Dentistry, Çankırı Karatekin University, Çankırı, Turkey. Performed the statistical analyses, contributed to the interpretation of the data, and critically revised the manuscript. Reviewed and approved the final version of the manuscript.; c Başak Karasu Assistant Professor, Department of Periodontology, Faculty of Dentistry, Çankırı Karatekin University, Çankırı, Turkey. Performed the periodontal examinations, participated in data collection, contributed to data interpretation, and critically revised the manuscript for important intellectual content. Reviewed and approved the final version of the manuscript.

**Keywords:** halitosis, menstrual cycle, oral malodour, salivary flow rate, whole-saliva pH

## Abstract

**Background:**

Hormonal fluctuations during the menstrual cycle may influence oral conditions, including halitosis and whole-saliva pH characteristics. However, the relationship between menstrual cycle phases, halitosis, salivary flow rate, and salivary pH has not been fully elucidated.

**Methods and Materials:**

This prospective observational study included 32 systemically healthy female dental students aged 18–25 years with regular menstrual cycles. Measurements were performed during three phases of the menstrual cycle: menstrual (days 1–4), follicular (days 7–11), and luteal (days 24–27). Halitosis was assessed using both organoleptic evaluation and a portable device (Breath Checker®). Salivary flow rate was measured using the spitting method, and whole-saliva pH was determined with a digital pH meter. Clinical oral parameters, including plaque-positive surfaces (%) and bleeding on probing, were also recorded. Data were analysed using the Friedman test and Spearman’s correlation analysis.

**Results:**

No statistically significant differences were observed in halitosis scores, salivary flow rate, plaque-positive surfaces (%), or bleeding on probing across menstrual cycle phases (*p* > 0.05). Whole-saliva pH showed a significant difference among phases (*p* = 0.017), with higher values in the menstrual phase compared to the luteal phase (*p* = 0.006). A significant positive correlation was found between salivary flow rate and salivary pH (rho = 0.511, *p* < 0.001).

**Conclusion:**

Menstrual cycle phases did not significantly affect halitosis or salivary flow rate in systemically healthy women. However, whole-saliva pH varied significantly, suggesting that hormonal fluctuations may influence the biochemical properties of saliva rather than its quantity. These findings highlight the importance of considering hormonal effects when interpreting salivary parameters in clinical practice.

Halitosis, defined as an unpleasant odour emanating from the oral cavity, is a common condition frequently referred to as bad breath or oral malodour.^10,26
^ It affects a considerable portion of the population and represents one of the most frequent complaints in dental practice, following dental caries and periodontal diseases.^15,22
^ While its aetiology is multifactorial, encompassing both intraoral and extraoral causes, most cases are primarily associated with intraoral factors such as tongue coating, periodontal disease, and poor oral hygiene.^10,11,15
^ Physiological halitosis may also occur transiently as ‘morning breath’, which is characterised by increased oral malodour upon awakening and has been associated with reduced salivary flow during sleep.^23^ Recent evidence further indicates that lower unstimulated salivary flow is associated with higher volatile sulphur compound levels and greater morning oral malodour, supporting the contribution of reduced salivary secretion to this physiological phenomenon.^17^


Volatile sulphur compounds (VSCs) are the primary compounds responsible for oral malodour, with hydrogen sulphide, methyl mercaptan, and dimethyl sulphide playing a key role in this process.^5^ These compounds are produced by anaerobic Gram-negative oral microorganisms through the degradation of sulphur-containing substrates such as food debris, saliva, blood, and epithelial cells present in the oral cavity.^12^


In females, physiological fluctuations in sex hormones, particularly oestrogen and progesterone, during the menstrual cycle may have various effects on the oral cavity.^14^ These hormonal changes may enhance inflammatory responses in gingival tissues, alter the susceptibility of periodontal tissues to bacterial challenges, and influence the balance of oral microorganisms.^8^ Additionally, hormonal fluctuations may lead to changes in salivary flow rate and pH, thereby affecting the biochemical balance of the oral environment.^4,20
^ These alterations may modulate the production of VSC and contribute to the development and severity of halitosis.^6,9,24
^


Although several studies have evaluated the effects of the menstrual cycle on oral conditions, the findings regarding its impact on halitosis are variable across studies. While some studies have reported increased levels of VSC during certain phases of the cycle,^6,9
^ a study has reported no significant changes in halitosis across menstrual cycle phases.^16^ In addition, hormonal fluctuations have been shown to influence salivary flow rate and pH, which play a key role in maintaining oral homeostasis.^21^ However, studies evaluating halitosis together with salivary flow rate and pH are limited. Furthermore, the potential association between hormonal fluctuations, salivary parameters, and halitosis has not been comprehensively elucidated, as these variables have rarely been assessed concurrently within the same study design. A better understanding of these physiological variations may help determine whether menstrual cycle phase should be considered when interpreting halitosis and salivary measurements in young women. This may also help distinguish normal cycle-related variations in the oral environment from changes that may warrant further clinical evaluation. Therefore, the relationship between hormonal changes and these clinical parameters during the menstrual cycle remains to be further clarified. The aim of this study was to evaluate changes in halitosis severity, salivary flow rate, and whole-saliva pH across different phases of the menstrual cycle in female individuals. The null hypothesis was that there would be no significant differences in halitosis, salivary flow rate, whole-saliva pH, percentage of plaque-positive surfaces, or bleeding on probing across the different phases of the menstrual cycle.

## Methods and Materials

The study was approved by the Çankırı Karatekin University Health Sciences Ethics Committee (Approval No: 2025/11/14–23) and conducted in accordance with the principles of the Declaration of Helsinki. Prior to participation, written informed consent was obtained from all individuals.

### Study Design and Participants

This prospective observational study was conducted among female students aged 18–25 years at Çankırı Karatekin University Faculty of Dentistry. Dental students were recruited to ensure a relatively homogeneous population with good oral hygiene and to improve adherence to pre-measurement protocols, such as dietary restrictions and avoidance of confounding factors (e.g. perfume, smoking, and oral hygiene practices). Initially, 34 participants were recruited; however, 2 participants were excluded due to irregular menstrual cycles. Thus, a total of 32 participants with regular menstrual cycles were included in the study.

Inclusion criteria were as follows: absence of systemic diseases that could cause halitosis, such as uncontrolled diabetes mellitus, gastroesophageal reflux disease, hepatic or renal insufficiency, respiratory tract infections, chronic sinusitis, or tonsillitis; good oral hygiene and absence of active gingival or periodontal disease requiring treatment; no use of antibiotics or other antimicrobial agents within the previous month; no use of medications that may influence oral malodour, such as antiseptic mouthrinses, proton pump inhibitors, antihistamines, antidepressants, or xerostomia-inducing drugs; non-smoking status; and no ongoing orthodontic treatment. Additionally, only participants with regular menstrual cycles (25–35 days) who were non-pregnant and had not used oral contraceptives within the previous 6 months were included. All participants were informed about the study protocol, and written informed consent was obtained prior to participation.

### Study Protocol

Menstrual cycle phases were determined according to self-reported menstrual history and cycle length. Only participants with regular menstrual cycles (25–35 days) were included in the study. Data were collected during three different phases of the menstrual cycle:

Menstrual phase (days 1–4)Follicular phase (days 7–11)Luteal phase (days 24–27)

All measurements were performed in the morning hours under standardised clinical conditions. Participants were instructed to refrain from eating, drinking (except water), toothbrushing, mouthwash use, smoking, and consumption of garlic, onion, alcohol, or coffee for at least 12 h prior to the measurements. Additionally, participants were asked not to drink water, use perfume, or apply scented products within 1 h before the measurements.

### Halitosis Assessment

Halitosis was evaluated using both organoleptic assessment and a portable device (Breath Checker®, Tanita Corp., Tokyo, Japan).

For organoleptic assessment, participants were seated upright and asked to keep their mouths closed for 3 min before gently exhaling. The examiner evaluated the odour at a distance of approximately 10 cm and scored it according to Rosenberg’s scale (0–5).^19^ To ensure reliability, organoleptic assessments were performed by two calibrated examiners. Prior to the study, calibration sessions were conducted, and interexaminer agreement was confirmed. The final score was recorded as the mean of the two measurements.

Organoleptic scores were recorded as follows:

0: No odour1: Barely noticeable odour2: Slight but clearly noticeable odour3: Moderate odour4: Strong odour5: Extremely strong odour

The Breath Checker® device (Tanita, Tokyo, Japan) was prepared according to the manufacturer’s instructions and allowed to stabilise before each measurement session. All measurements were performed under standardised conditions by the same examiner (S.A.). Participants were instructed to keep their mouths closed for 3 min prior to the assessment. The device was activated by opening the upper cover, and after the countdown from 5 and the appearance of the ‘START’ signal, it was positioned approximately 1 cm from the participant’s mouth. Participants were then instructed to exhale toward the sensor until a beep sound was emitted (approximately 5 s), and the displayed odour level was recorded.

The scores were interpreted as follows:

0: No odour1: Slight odour2: Moderate odour3: Noticeable odour4: Strong odour5: Very strong odour

### Salivary Flow Rate and pH Measurement

Saliva samples were collected using the ‘spitting method’ as previously described.^25^ Before the collection procedure, participants were instructed to swallow any existing saliva in the mouth. Subsequently, saliva was allowed to accumulate passively in the floor of the mouth without stimulation of salivary secretion by orofacial movements. The accumulated saliva was collected over a period of 5 min, and the total volume was measured. Salivary flow rate was then calculated and expressed in ml/min.

The pH of pooled whole saliva was measured using a digital pH meter (Hanna HI 2211, Hanna Instruments, Woonsocket, RI, USA). As the samples represented whole-saliva accumulated in the oral cavity rather than saliva collected directly from individual salivary gland ducts, the measured pH was considered to reflect the combined oral environment represented in pooled whole saliva. The electrode was rinsed with distilled water before each measurement and then immersed into the saliva sample. The reading was recorded once a stable value was obtained. The device was calibrated using standard buffer solutions according to the manufacturer’s instructions prior to measurements.

### Clinical Oral Examination

Dental plaque accumulation and bleeding on probing (BOP) were assessed for all teeth in the four quadrants. All clinical measurements were performed twice by a calibrated periodontist (B.K.) using a Williams periodontal probe (Hu-Friedy, Chicago, IL, USA) marked in millimetres. For plaque assessment, four surfaces of each tooth (mesial, distal, buccal, and lingual/palatal) were examined using a periodontal probe without a plaque-disclosing agent. Plaque presence was recorded dichotomously as present (1) or absent (0). The percentage of plaque-positive surfaces was calculated by dividing the number of plaque-positive surfaces by the total number of examined surfaces and multiplying by 100.

Gingival inflammation was evaluated using BOP.^1^ Periodontal probing was performed with a standardised force of approximately 0.25 N at six sites per tooth (mesiobuccal, buccal, distobuccal, mesiolingual, lingual, and distolingual). Bleeding occurring within 10 s after probing was recorded as present (1) or absent (0). The percentage of BOP was calculated by dividing the number of bleeding sites by the total number of examined sites and multiplying by 100. As all participants met the eligibility criteria for gingival and periodontal health and none required periodontal treatment at baseline, no periodontal intervention was performed before the observational period. Plaque-positive surfaces (%) and BOP were recorded at each menstrual cycle phase to monitor oral hygiene and gingival inflammatory status throughout the study.

### Statistical Analysis

The primary outcome of the study was halitosis score, while salivary flow rate, whole-saliva pH, plaque-positive surfaces (%), and BOP were considered secondary outcomes. Statistical analyses were performed using the R programming language (version 4.4.3) within the RStudio integrated development environment (version 2026.1.0.392). The normality of the data distribution was assessed using the Shapiro–Wilk test. As the data did not follow a normal distribution, non-parametric methods were applied.

As no formal *a priori* sample size calculation was performed prior to recruitment, a sensitivity power analysis was conducted using the WebPower package in R. With 32 participants, three repeated measurements, an α level of 0.05, and 80% power, the study had 80% power to detect within-subject effects of Cohen’s f ≥ 0.56.

Descriptive statistics were presented as median and interquartile range (IQR). Differences among the follicular, luteal, and menstrual phases were evaluated using the Friedman test for repeated measures. Effect sizes were calculated using Kendall’s W coefficient.

For variables showing significant differences or trends toward significance, post-hoc pairwise comparisons were performed using the Wilcoxon signed-rank test with Bonferroni correction.

Spearman’s rank correlation coefficient was used to assess the relationships between whole-saliva pH, salivary flow rate, and organoleptic scores across menstrual phases. The agreement between the two halitosis assessment methods was assessed using Spearman’s rank correlation between organoleptic scores and Breath Checker readings. All statistical analyses were conducted using the rstatix and ggplot2 packages. A *p* value < 0.05 was considered statistically significant.

## Results

A total of 32 participants completed all study phases and were included in the final analysis.

Descriptive statistics of all clinical parameters across the follicular, luteal, and menstrual phases are presented in Table 1.

**Table 1 Table1:** Clinical parameters across menstrual cycle phases

Clinical parameters	Follicular phase med (IQR)	Luteal phase med (IQR)	Menstrual phase med (IQR)	*p* value (Friedman)	Kendall’s W
Bleeding on probing (%)	3.14 (1.78–9.24)	4.57 (1.78–10.25)	5.35 (3.6–8.93)	0.082	0.08
Breath Checker score	1 (0–1)	1 (0–2)	1 (0.75–2.25)	0.055	0.09
Organoleptic score	1 (0–1)	1 (0–1)	1 (1–2)	0.062	0.09
Whole-saliva pH	7.34 (7.02–7.55)	7.36 (6.92–7.52)	7.47 (7.22–7.77)	0.017	0.13
Plaque-positive surfaces (%)	15.17 (10.27–34.95)	19.64 (9.06–30.45)	15.17 (8.92–25.89)	0.535	0.02
Salivary flow rate (mL/min)	2.1 (1.28–2.68)	2 (1.28–2.83)	2.2 (1.2–2.78)	0.968	0
Med (IQR) = Median (25th–75th Percentiles). n = 32.					

The Friedman test revealed a statistically significant difference in whole-saliva pH across the menstrual cycle phases (*p* = 0.017, Kendall’s W = 0.13). Post-hoc analysis using the Wilcoxon signed-rank test with Bonferroni correction showed that whole-saliva pH was significantly higher during the menstrual phase compared to the luteal phase (*p* = 0.006). No significant differences were observed between the follicular and luteal phases (*p* = 0.306) or between the follicular and menstrual phases (*p* = 0.375).

No statistically significant differences were found among the menstrual cycle phases for bleeding on probing (*p* = 0.082), plaque-positive surfaces (%) (*p* = 0.535), or salivary flow rate (*p* = 0.968).

Breath measurements (*p* = 0.055) and organoleptic scores (*p* = 0.062) demonstrated a trend towards significance; however, these differences did not reach statistical significance after correction.

Spearman correlation analysis revealed a significant positive correlation between salivary flow rate and whole-saliva pH during the luteal (rho = 0.609, *p* < 0.001) and menstrual phases (rho = 0.581, *p* < 0.001), as well as in the overall dataset (rho = 0.511, *p* < 0.001). These relationships are illustrated in Figure 2. No significant correlations were found between organoleptic scores and whole-saliva pH or salivary flow rate in any phase (*p* > 0.05). A strong positive correlation was observed between organoleptic scores and Breath Checker measurements across all cycle phases (follicular rho = 0.69, luteal rho = 0.74, menstrual rho = 0.61; overall rho = 0.67, *p* < 0.001), supporting the concurrent validity of the two assessment methods. The sensitivity analysis indicated that, with 32 participants, the study had 80% power to detect within-subject effects of Cohen’s f ≥ 0.56. Thus, the achieved sample size allowed detection of relatively large within-subject effects, whereas smaller effects may have remained undetected.

**Fig 2 Fig2:**
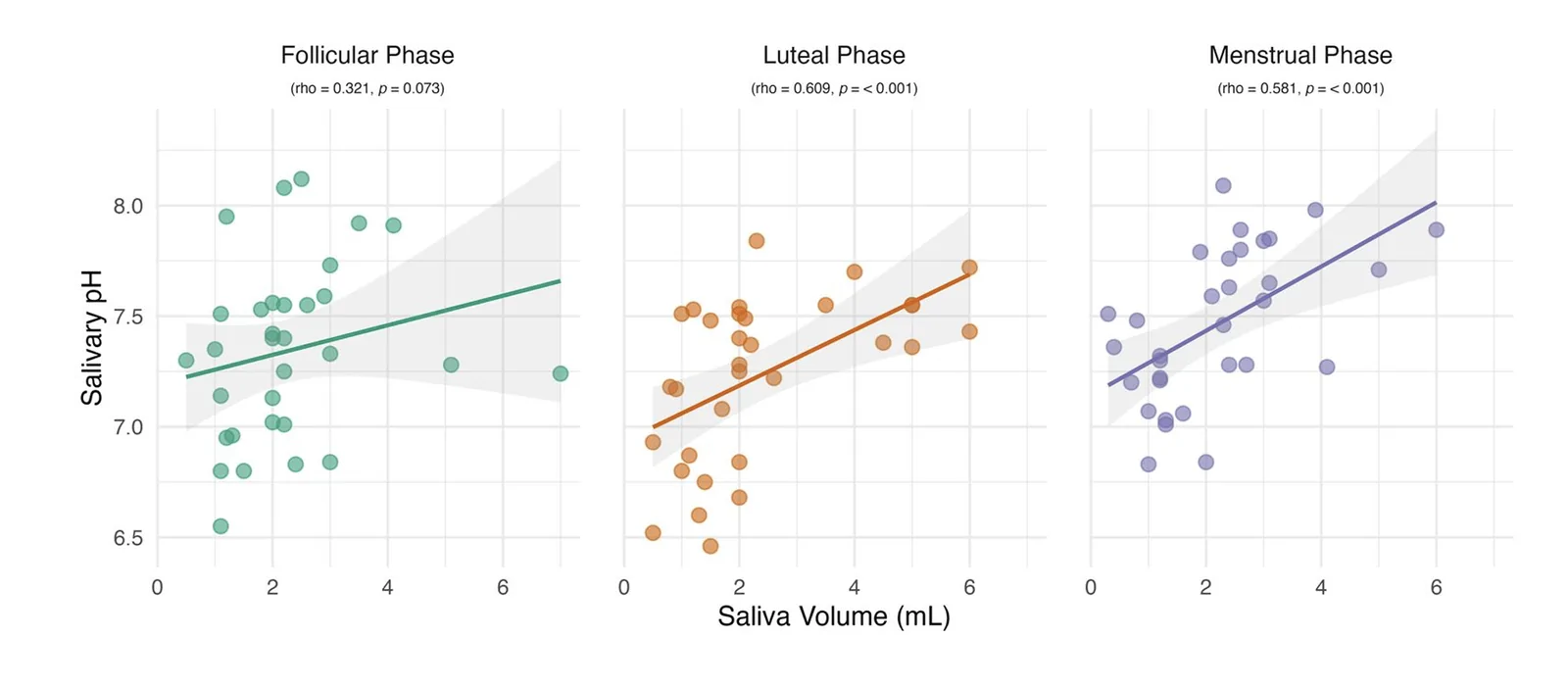
Correlation between salivary flow rate and whole-saliva pH across menstrual cycle phases. Scatter plots show the relationship between salivary flow rate (mL/min) and whole-saliva pH during the follicular, luteal, and menstrual phases. Spearman correlation coefficients (rho) and corresponding *p* values are presented for each phase.

The distribution of clinical measurements across the menstrual cycle phases is shown in Figure 1, whereas the correlations between salivary flow rate and whole-saliva pH are shown in Figure 2.

**Fig 1 Fig1:**
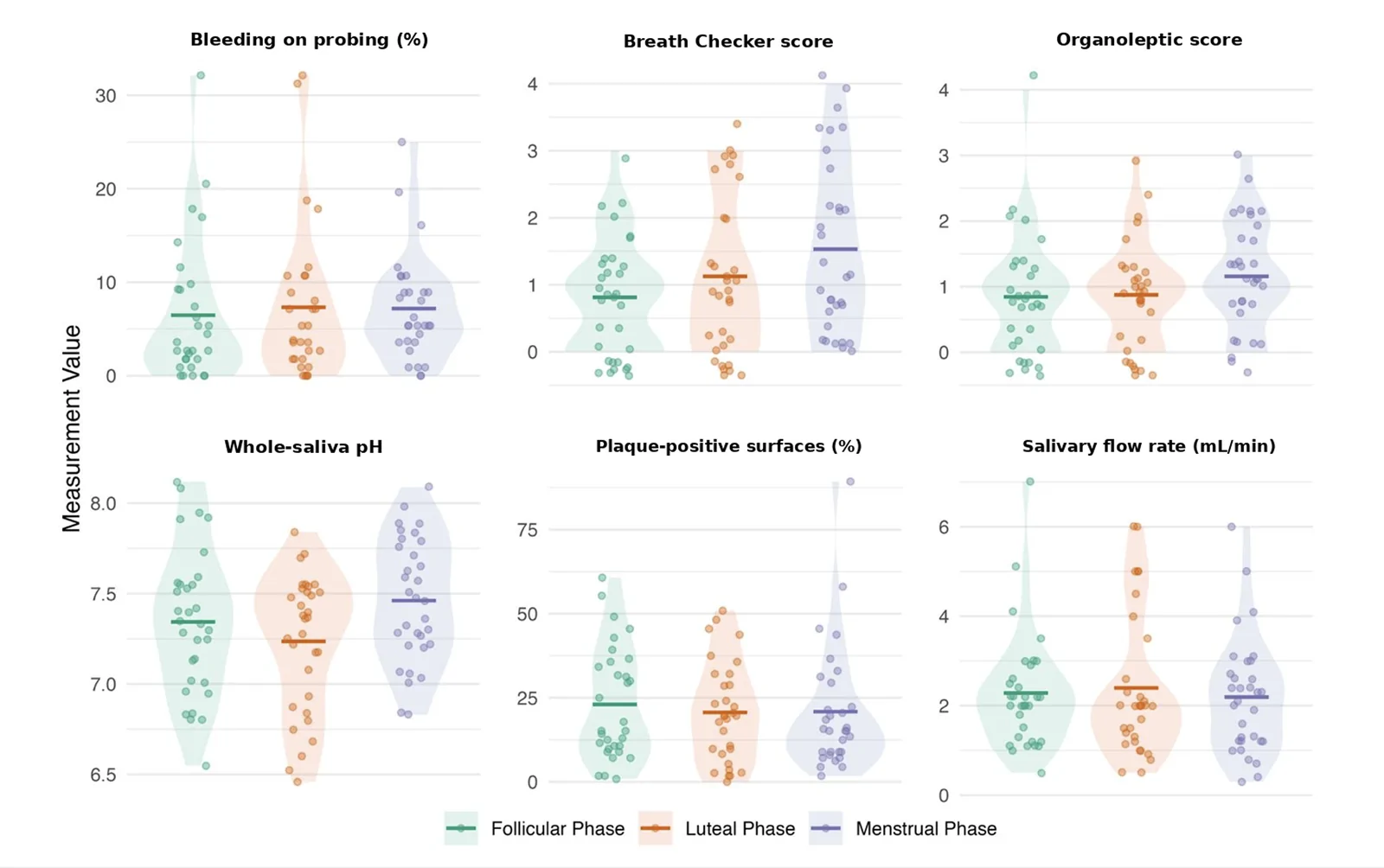
Distribution of clinical parameters and their relationships across menstrual cycle phases. Violin plots illustrate the distribution of bleeding on probing (%), Breath Checker scores, organoleptic scores, whole-saliva pH, plaque-positive surfaces (%), and salivary flow rate during the follicular, luteal, and menstrual phases. Data are presented as median and interquartile range (IQR).

## Discussion

The present study aimed to evaluate the effect of menstrual cycle phases on halitosis, salivary flow rate, and whole-saliva pH in systemically healthy young women. The findings of this study demonstrated that halitosis levels, assessed using both organoleptic evaluation and a portable device, did not show significant differences across the menstrual cycle phases. Similarly, salivary flow rate remained stable throughout the cycle. In contrast, whole-saliva pH exhibited significant variation, with lower values observed during the luteal phase. In addition, a significant positive correlation was found between salivary flow rate and whole-saliva pH. This association suggests that variations in salivary secretion may contribute to changes in the overall oral environment, rather than reflecting intrinsic biochemical changes in glandular saliva alone.

In the literature, halitosis across different phases of the menstrual cycle has been evaluated using both objective methods measuring VSCs^6,9
^ and questionnaire-based studies relying on self-reported oral malodour.^2,3,18
^ However, a review of the literature indicates that studies employing organoleptic assessment, which is considered the gold standard^5^ despite its subjective nature, are limited, whereas studies using device-based measurement methods are more common.^6,9,16
^ The findings of these studies are not entirely consistent. Calil et al reported^6^ a statistically significant increase in VSC levels during the premenstrual and menstrual phases, while Kakoei et al demonstrated^9^ higher VSC levels in the menstrual phase compared to the follicular and premenstrual phases. In contrast, a previous study reported no significant differences in halitosis across menstrual cycle phases when assessed using both organoleptic and device-based methods.^16^ In this context, the available evidence suggests that the effects of hormonal fluctuations on halitosis remain inconclusive. The findings of the present study showed no statistically significant differences in halitosis across the evaluated menstrual cycle phases; however, given the sensitivity of the study to relatively large effects, smaller potentially clinically relevant differences cannot be excluded.

When the studies investigating salivary flow rate across different phases of the menstrual cycle are reviewed, one study^6^ reported a decrease in salivary flow rate during the premenstrual and menstrual phases, whereas another study^9^ demonstrated no significant differences in salivary flow rate across menstrual cycle phases and no significant association between salivary flow rate and halitosis. Similarly, the findings of the present study showed no significant differences in salivary flow rate across the menstrual cycle phases. The lack of significant differences in salivary flow rate across menstrual cycle phases may reflect physiological regulatory mechanisms that maintain relatively stable salivary secretion; however, the present study did not directly assess hormonal changes underlying these phases. Furthermore, variations in study design, sample size, and saliva collection methods may contribute to the inconsistent findings reported in the literature.

In addition, although several studies have evaluated salivary flow rate and salivary pH during periods characterised by intense hormonal fluctuations, such as the menstrual cycle, pregnancy, and menopause^13,21,27
^ studies specifically investigating salivary pH across different phases of the menstrual cycle remain limited.^4^ Available evidence suggests that salivary pH tends to decrease during the premenstrual phase and that this change may be associated with hormonal fluctuations.^27^ In line with these findings, the present study also demonstrated a significant decrease in whole-saliva pH during the luteal phase. The observed variation in whole-saliva pH may reflect changes in the overall oral environment across menstrual cycle phases rather than intrinsic biochemical changes in glandular saliva alone. Since pH was measured in pooled whole saliva rather than saliva collected directly from individual salivary gland ducts, the observed differences may reflect the combined influence of salivary secretion and other components of the oral environment. Moreover, the significant positive correlation observed between salivary flow rate and whole-saliva pH suggests that variations in salivary secretion may contribute to the observed changes in the oral environment.

The percentage of plaque-positive surfaces and BOP values did not differ significantly across menstrual cycle phases, indicating that oral hygiene and gingival inflammatory status remained relatively stable throughout the observation period. These parameters were monitored as potential confounding factors when evaluating changes in halitosis and whole-saliva pH across the menstrual cycle.

In the present study, halitosis was evaluated using both organoleptic assessment and a portable device (Breath Checker®) to minimise the limitations associated with subjective self-reports. Although various device-based methods have been developed to assess oral malodour, the use of simple portable devices (Breath Checker®) has been less frequently reported in the literature.^7,28
^ A previous study reported a moderate correlation between Breath Checker measurements and organoleptic scores (r = 0.404).^28^ Similarly, the present study demonstrated significant positive correlations between the two methods across all menstrual cycle phases, with correlation coefficients ranging from 0.61 to 0.74. These findings further support the use of the Breath Checker as a practical adjunct to organoleptic assessment. However, such devices have certain limitations, including the inability to provide compound-specific measurements and their susceptibility to environmental factors.^7^ The portable monitor provides a semi-quantitative assessment of oral malodour based on the detection of volatile compounds in exhaled air. However, it does not allow direct quantification of individual VSCs or identification of specific microbiological contributors to oral malodour. Therefore, while the combined use of the Breath Checker and organoleptic assessment may have contributed to a more comprehensive evaluation of halitosis, these limitations should be considered when interpreting the findings.

### Limitations

This study has some limitations that should be considered when interpreting the findings. First, halitosis was evaluated using a portable device rather than advanced analytical instruments such as gas chromatography or halimeters, which may provide more detailed compound-specific measurements. Second, microbiological analyses were not performed; therefore, the potential relationship between oral microbial composition and halitosis could not be assessed.

In addition, menstrual cycle phases were determined according to self-reported menstrual history and calendar-based timing rather than hormonal confirmation. Therefore, the possibility of phase misclassification cannot be completely excluded. Importantly, tongue coating, which is recognised as one of the principal intraoral sources of halitosis, was not specifically assessed during the clinical examination. Given the primary focus of the present study on halitosis, the absence of tongue-coating assessment represents an important limitation, as its potential contribution to the observed oral malodour could not be accounted for. Although the participants had good oral hygiene and low levels of plaque accumulation and gingival inflammation, these parameters do not exclude the possible influence of tongue coating on halitosis.

The study population consisted of systemically healthy young female dental students with good oral hygiene, low levels of plaque accumulation and gingival inflammation, and low baseline halitosis levels. While this homogeneity minimised potential confounding factors, it may also have limited the ability to detect subtle differences in halitosis across menstrual cycle phases. Moreover, the restricted characteristics of the study population limit the generalisability of the findings. Therefore, the results may not be directly generalisable to women with poorer oral hygiene, periodontal disease, systemic conditions, smokers, or the broader female population.

Finally, the sample size and the evaluation of a single menstrual cycle may be considered additional limitations. A post-hoc sensitivity analysis indicated that the achieved sample of 32 participants provided 80% power to detect relatively large within-subject effects (Cohen’s f ≥ 0.56). Consequently, small or moderate effects may have remained undetected, and the absence of statistically significant differences in halitosis and other outcomes should not be interpreted as evidence of the absence of potentially clinically relevant differences across menstrual cycle phases. Measurements were obtained once during each selected menstrual cycle phase, and repeated measurements within the same phase or across multiple menstrual cycles were not performed. Such repeated assessments could have better characterised intra-individual variability and provided a more robust estimate of cycle-related changes. Further studies including larger sample sizes, repeated measurements within each phase, assessments across multiple menstrual cycles, hormonal confirmation of menstrual phases, and more comprehensive analytical methods are needed to better elucidate the relationship between hormonal fluctuations, salivary characteristics, and oral malodour.

## Conclusion

Within the limitations of this study, halitosis scores and salivary flow rate did not differ significantly across menstrual cycle phases in systemically healthy female dental students with good oral hygiene. However, whole-saliva pH showed a statistically significant, albeit modest, variation, with higher values during the menstrual phase compared with the luteal phase. These findings suggest that menstrual cycle-related variations in the oral environment may warrant consideration when interpreting whole-saliva pH measurements. Given the specific characteristics of the study population, these findings should be interpreted cautiously and may not be generalisable to the broader female population. Further studies involving larger and more diverse populations, with hormonally confirmed menstrual cycle phases, are needed.

### Acknowledgements

#### Funding

The authors received no specific financial support for the research, authorship, or publication of this article.

#### Conflict of interest

The authors declare no conflicts of interest related to this study.

#### Ethics approval

The study was approved by the Çankırı Karatekin University Health Sciences Ethics Committee (Approval No: 2025/11/14–23) and was conducted in accordance with the Declaration of Helsinki. Written informed consent was obtained from all participants prior to enrolment.

#### AI use statement

Generative artificial intelligence (ChatGPT, OpenAI) was used solely to assist with language editing, grammar correction, and improvement of manuscript readability. No artificial intelligence tools were used for data collection, data analysis, statistical analysis, interpretation of results, or generation of scientific conclusions. All content was critically reviewed and approved by the authors, who take full responsibility for the final manuscript.

## References
